# Artificial intelligence prediction of the effect of rehabilitation in whiplash associated disorder

**DOI:** 10.1371/journal.pone.0243816

**Published:** 2020-12-17

**Authors:** Alberto Javier Fidalgo-Herrera, María Jesús Martínez-Beltrán, Julio Cesar de la Torre-Montero, José Andrés Moreno-Ruiz, Gabor Barton

**Affiliations:** 1 Biomechanics department, Fisi(ON) Health Group, RetiaTech, Las Rozas, Spain; 2 San Juan de Dios School of Nursing and Physical Therapy, Comillas Pontifical University, Madrid, Spain; 3 Departament of Computing, University of Almería, Almería, Spain; 4 School of Sport and Exercise Sciences, Liverpool John Moores University, Liverpool, United Kingdom; University of Pittsburgh, UNITED STATES

## Abstract

The active cervical range of motion (aROM) is assessed by clinicians to inform their decision-making. Even with the ability of neck motion to discriminate injured from non-injured subjects, the mechanisms to explain recovery or persistence of WAD remain unclear. There are few studies of ROM examinations with precision tools using kinematics as predictive factors of recovery rate. The present paper will evaluate the performance of an artificial neural network (ANN) using kinematic variables to predict the overall change of aROM after a period of rehabilitation in WAD patients. To achieve this goal the neck kinematics of a cohort of 1082 WAD patients (55.1% females), with mean age 37.68 (SD 12.88) years old, from across Spain were used. Prediction variables were the kinematics recorded by the EBI^®^ 5 in routine biomechanical assessments of these patients. These include normalized ROM, speed to peak and ROM coefficient of variation. The improvement of aROM was represented by the Neck Functional Holistic Analysis Score (NFHAS). A supervised multi-layer feed-forward ANN was created to predict the change in NFHAS. The selected architecture of the ANN showed a mean squared error of 308.07–272.75 confidence interval for a 95% in the Monte Carlo cross validation. The performance of the ANN was tested with a subsample of patients not used in the training. This comparison resulted in a medium correlation with R = 0.5. The trained neural network to predict the expected difference in NFHAS between baseline and follow up showed modest results. While the overall performance is moderately correlated, the error of this prediction is still too large to use the method in clinical practice. The addition of other clinically relevant factors could further improve prediction performance.

## Introduction

Classically, whiplash is defined as an acceleration–deceleration mechanism of energy transferred to the neck resulting from rear-end or side-impact motor vehicle collisions, but also from diving or other mishaps. These injury mechanisms can cause what is known as whiplash associated disorder (WAD). Common signs and symptoms of WAD include neck pain, stiffness and headaches [1,2]. The cost associated with this condition is EUR 10 billion per annum in Europe alone [3]. The incidence of this pathology varies across countries, but the average is 300 cases per 100000 inhabitants [4,5]. Recovery rates have not improved over the past decades with 50% of patients maintaining symptoms 6 months post injury and 20% not being considered recovered 5 years after the injury [6–11]. WAD has a clear impact on health-related quality of life (HRQoL), and the worse the level of disability, the lower the HRQoL and the higher the costs for society [12].

This burden for the health system has made many clinicians and researchers to turn their attention into WAD to get a better understanding of the pathology. The available evidence suggests that cervical range of motion impairment is mainly caused by pain [13]. While active cervical range of motion (aROM) is routinely assessed by clinicians, this assessment is often performed using unstandardized subjective methods [14]. A recent systematic review on the clinical tests used to evaluate cervical pathologies stated that visual inspection and palpation are inconsistent measures for the problems found in cervical pathologies. In this regard, both visual inspection and palpation showed low inter-rater reliability scores and therefore other methods of evaluating cervical pathology are suggested [15]. Some attempts have been made to standardize the evaluation of the cervical spine, such as validating the use of goniometry [16,17] or tape measures [18]. However, these methods are unable to address other parameters of the movement such as the speed or detect subtle changes in ROM. Advances in inertial measurement unit (IMU) technology have simplified data acquisition by making available devices that are accurate, cheap and easy to use and can objectively measure aROM [19–23].

Using equivalent systems Grip et al. [24] explored the idea of differentiating WAD patients from healthy controls using only the neck kinematics. In their study, multiple parameters are used to train a neural network. While their results were promising, a sample of 56 controls and 59 patients was deemed small to train a generalizable neural network. It is noteworthy that the authors point out that speed parameters show most differences between injured and healthy subjects and therefore they were the principal regressors that defined the group assignment [25]. These conclusions are further shared by researchers such as Niederer et al. [26]. Recently published work expanded the identification of presence or absence of injury by determining the stages of injury in cervical pathologies. This work presented a novel measure, the NFHAS (Neck Functional Holistic Analysis Score). This score encompasses all ranges of motion and assigns a single value for the cervical mobility. With the NFHAS the cervical impairment was staged in 5 categories of increasing severity, easing the identification of the seriousness of the injury according to kinematics [27].

Even with the ability of neck motion to discriminate injured from non-injured subjects, the mechanisms to explain recovery or persistence of WAD remain unclear [28]. The development of clinical prediction rules (CPRs) came about to help in the identification of patients at risk of developing chronic WAD. These tools are designed to guide the clinicians’ decisions [7,29]. The whiplash CPR uses the neck disability index (NDI), the posttraumatic diagnostic scale and the age of the patient [8]. The NDI considers the aROM of the patient but with a subjective approach. Since the CRP has shown good overall performance, the aROM measured objectively could improve the performance of prediction. A major systematic review and meta-analysis of cohort studies identified that there is a lack of knowledge on hard physical objective signs that can act as predictors of the most probable progression of the pathology [1]. Further, it is reported that the procedures used to study WAD are highly inconsistent and this makes it difficult to compare and synthesize the literature in this area [30].

Synthesizing the reviewed literature, we hypothesize that a combination of aROM parameters could help determining the expected recovery of WAD patients. More severe injuries will take longer to recover than milder ones and therefore baseline kinematic parameters could identify this behaviour. To predict new values, regression approaches constitute the classical method. Prediction and explanation are two of the main applications of multiple regression [31], however, in recent years artificial neural networks (ANNs) have gained ground over this classic approach. In these regard la Delfa et al. [32] propose that ANNs are used more often in biomechanics as they appear to be the most robust method for predicting complex relationships. The name artificial neural network derivates from the similarities between the functioning of these algorithms and actual neurons from the nervous system. A neural network is composed of three main parts: the input layer, the hidden layers, and the output layer. The input layer is composed of as many nodes (neurons) as variables are used as predictors of the output. The output layer is composed of several nodes (neurons) equal to the number of target variables, the ones that are to be predicted. In the middle the hidden layers are found. The configuration for these hidden layers is not standardized, and their number or the number of neurons to be included remains to the choice of the investigator [33]. Some authors suggest that most of the problems, where ANNs are used on, can be solved with just one single hidden layer. It is also suggested that the number of neurons in the hidden layers should range between the number of neurons between the input and the output layers [34]. All these layers are connected to each other like neurons in the nervous system are connected by their axons. These connections have a bias and a weight associated that condition when a neuron is activated. All these weight and biases are automatically adjusted by optimization algorithms. These algorithms adjust the weights and biases depending on a correct or incorrect prediction of the target variable during the training phase of the ANN. In a nutshell, an ANN acts as a compilation of regression analyses that work together to make a prediction.

The aim of this paper was to evaluate the performance of a neural network model using kinematic variables to predict the overall change of aROM after a period of rehabilitation in WAD patients.

## Materials and methods

### Study design and population

A retrospective approach was used to develop a neural network capable of predicting the direction and magnitude of change in the Neck Functional Holistic Analysis Score (NFHAS) after rehabilitation in WAD patients [27]. To achieve this goal the records of the neck kinematics of a cohort of WAD patients from across Spain were used. The WAD patients from traffic car collisions assessed by Fisi(ON) Health Group or its associated clinics were used for this research. All the network of professionals certified to do the assessments are uniformly trained by Fisi(ON) Health Group personnel and follow the same standardized and strict protocol. The measuring device used in these assessments is the EBI^®^ 5, which uses two IMUs for the kinematics recordings at a certified precision of ±0.1°. The patient sits on an upright position, one IMU is placed upon the occiput with an elastic headband, and the other one over the space between the spinous processes of T2 and T3 with double-sided hypoallergenic adhesive tape. The evaluated patient performs an oscillatory movement between the ROM limits of each movement pair (flexion/extension, right side bend/left side bend, right rotation/left rotation) for 45 seconds each. The speed of movement is self-determined, and the patient has the goal to perform the maximum number of repetitions. For these movements, the angles between the head and the thoracic spine are calculated to get the range of motion of the cervical spine. All patients’ records were received in the central headquarters of Fisi(ON) for interpretation and reporting. Data collection was repeated for trials that contained unusable results followed by analysis. The inclusion criteria to be included in this study were: patients over 18 years old, having a diagnosis related to cervical spine injuries, have a baseline assessment before treatment and a follow up assessment after treatment between January and August 2019. All the patients received standard rehabilitation treatment for the neck between baseline and follow up. This treatment included manual therapy, exercises and electrotherapy. The exclusion criteria were: patients with any of the studied kinematic variables considered as an outlier, i.e. more than 3 scaled median absolute deviations away from the median [35]. All outliers were removed from the sample since those parameters could not correspond to real human kinematics. Those outliers could be a result of failed biomechanics assessments that were registered on the database. Few outliers were found since the system has control algorithms that prevent most of these happenings from being recorded. The work of Ogundimu et al. determined that at least 20 cases should be included for every variable used in a prediction model using binary predictors [36]. According to this rule of thumb it would be recommended to use at least 360 patients in this investigation. Since, our predictors are not binary and a larger dataset would reduce the risk of overfitting in the model [31], the full sample of 1082 patients will be used. Ethics approval was obtained from the Research Ethics Committee of *Hospital Clínico Universitario San Carlos*, Madrid, Spain (approval number 18/405-E). This organism is independent from any of the investigators to comply with the Good Clinical Practice guidelines. All the information used was obtained during routine care and was anonymized before its use in the current investigation. All patients had signed an informed consent where it was specified that clinical data could be used in clinical research.

#### Model design and statistical analysis

A supervised multi-layer feed-forward neural network was created to predict the change in NFHAS. The input layer is composed of 18 neurons corresponding to the predictors.

Predictor variables were derived from the kinematics recorded in the biomechanical assessments of the patients. The predictors included age normalized ROM of flexion (F), extension (E), left lateral bending (Llb), right lateral bending (Rlb), left rotation (Lrt) and right rotation (Rrt); coefficient of variation (CV) of each ROM; and speed to peak of each ROM. The ROM and speed were obtained averaging all the repetitions made during the recording. All variables were z-score normalized to avoid problems with the differences in the scale of measurement (i.e. percentage of movement, degrees per second).

To address the improvement of aROM the NFHAS was used. This measure combines the three normalized main ROMs (flexion-extension, lateral bending and rotations) giving a percentage of the global movement of the patient [37]. The procedure to obtain the NFHAS is as follows:

- Determine the sides of a polygon using ROM as coordinates
  $$D_{ROM_{a},ROM_{b}}=\sqrt{(x_{ROMa}-x_{ROMb}{)}^{2}+(y_{ROMa}-y_{ROMb}{)}^{2}+(z_{ROMa}-z_{ROMb}{)}^{2}}$$
  Where “a” and “b” are any 2 of the ROM with their “x”, “y” and “z” coordinates. The letter “D” represents the distance between 2 ROM vertices.
- Calculate the area with Heron’s formula:
  $$A=\sqrt{s(s-a)(s-b)(s-c)}$$
  Where a, b and c are the sides of the polygon, and s represents the semiperimeter calculated by:
  $$s=\frac{a+b+c}{2}$$
- The NFHAS is the result of comparting the area of this polygon against the ideal (100% in all ROM):
  $$NFHAS=\frac{A}{A_{100\%}}\times 100$$

The target variable was formed by subtracting the NFHAS obtained in the first assessment before rehabilitation from the NFHAS obtained in the assessment after rehabilitation.

$${NFHAS}_{difference}={NFHAS}_{2}-{NFHAS}_{1}$$

In previous investigations the NFHAS showed that 5 groups was the optimal number of clusters to stage the severity of ROM impairment [37].

The output layer was created using this the signed difference between pre- and post-rehabilitation NFHAS.

Some debate remains on the optimal architecture of ANNs. Most of the literature believes that just one hidden layer is enough to solve most of real-life problems. However, there is no definite answer on the optimal number of hidden layers and neurons in each layer [33,34]. Different architectures were tried practically and the one that offered the best performance was chosen. The levenberg-marquadt algorithm was used in the training for optimization of the ANN.

A Monte Carlo cross-validation was used to determine the optimal architecture of the ANN. A maximum of 17 neurons and 2 hidden layers were tested. At every epoch one neuron was added to the architecture or relocated to the second layer if testing a two-hidden-layer architecture. Seventy per cent of these were used for training and the thirty per cent remaining for validation and calculating the mean squared error (MSE) of the prediction. The MSE is the main variable to address the performance of ANNs. This metric shows the average magnitude of squares of error, the distance between the model’s estimate of the change in NFHAS and the actual change in NFHAs that each patient showed. Values closer to 0 demonstrate better performance and a more accurate forecasting of the ANN. Patients were randomly assigned to each group in each loop. For each possible combination, the MSE was obtained 10 times with different trained ANNs. The MSEs obtained were then averaged to obtain the effect on the error of selecting different cases for the training of the ANN.

The architecture that displayed the lowest MSE was chosen for the final training ANN. In this training patients were randomly assigned again to 2 groups. One group was used to train the ANN, the other group was used for validation purposes and was not presented to the ANN previously. To further test the precision of the ANN a regression using the predicted values of change in NFHAS as independent variable and the real change in NFHAS as dependent variable was performed. All the assumptions (linearity, normality, homoscedasticity, and independence) necessary to run a simple linear regression were satisfied, however, this model will not be used for prediction of measures but a measure of the performance of the developed ANN. An R value closer to 1 indicates a better fit between the regression model and the expected values.

Another ANN was trained adding principal component analysis (PCA) as pre-processing treatment to the predictor variables. This treatment would create new components that are unrelated to each other and potentially improve the prediction. The purpose of this PCA is to eliminate collinearity between the predictors, the maximum fraction of variance for removed rows was left on 0. Each of the PCA would be orthogonal to each other, therefore maximizing the differences between the input variables. Another linear regression analysis was conducted to assess performance, the assumptions for regression analysis were satisfied again.

The sample of patients was further divided into real improvement and predicted improvement (group 1 1), real improvement and predicted to not improve (group 1–1), real deterioration and predicted to improve (group -1 1) and real deterioration and predicted to not improve (group -1–1). A paired t-test was conducted between pre- and post-rehabilitation kinematic values in each group, significance level was set to alpha 0.05.

## Results

### Participants

A total of 1082 patients were included of which 55.1% were females and a 44.9% were males (age 37±12.88 years, height 1.67±0.09 m and mass 72.41±15.47 Kg). These patients were classified at baseline assessment according to the NFHAS in the following percentages: 3.69% NFHAS type 1, 16.82% NFHAS type 2, 24.67% NFHAS type 3, 31.23% NFHAS type 4 and 23.56% NFHAS type 5. The severity of the injury increases with the NFHAS group number, e.g. NFHAS type 1 describes complete ROM while NFHAS type 5 describes severe limitations.

**Fig 1:**
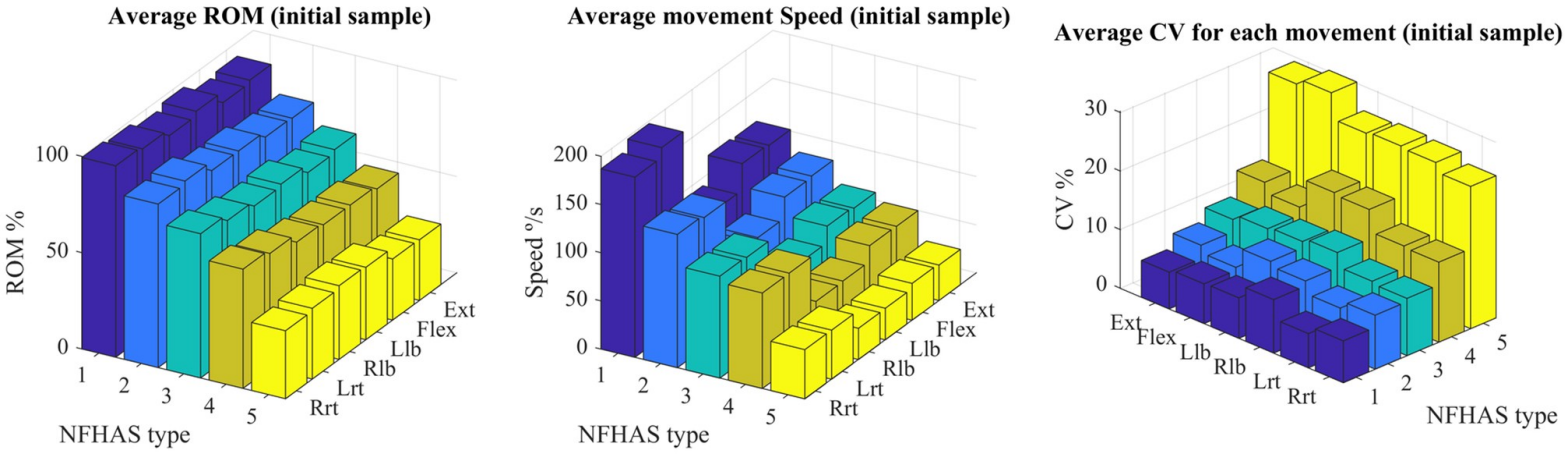
Baseline kinematic variables divided into severity of movement impairment according to NFHAS.

The ROM and the speed show a consistent decrease as the severity of injuries increases. The speed of the rotation is the highest followed by flexion-extension and last the lateral bending. This finding is consistent for all the NFHAS groups (Fig 1). The consistency of the movement decreases with increasing severity. The ROM CV is at its highest in the NFHAS type 5 group in all movements.

### ANN architecture selection

**Fig 2:**
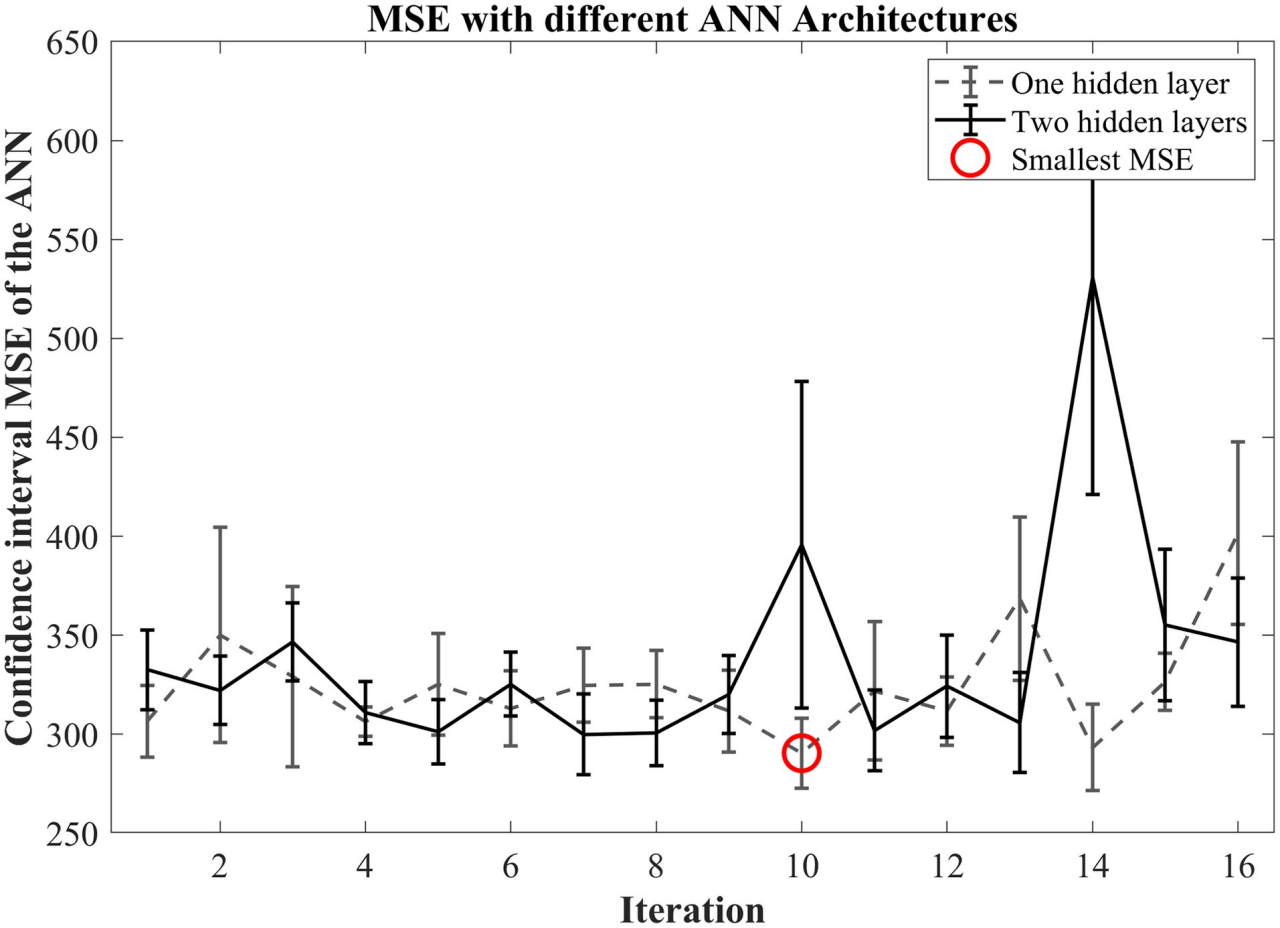
MSE average from the Monte Carlo cross-validation of different ANN architectures.

All the possible architectures of the ANN were tested with a Monte Carlo cross-validation method. This procedure showed that the MSE was the lowest with only one hidden layer with ten neurons. The mean MSE for this architecture was 290 with a confidence interval of 308.07–272.75 for a 95% confidence (Fig 2).

**Fig 3:**
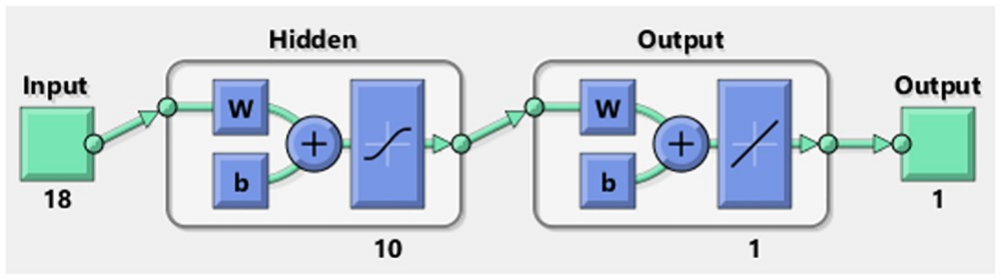
Final architecture of the ANN.

The resulting ANN was defined with an input layer of 18 neurons, a hidden layer with 10 neurons and an output layer with one regression output (Fig 3).

**Fig 4:**
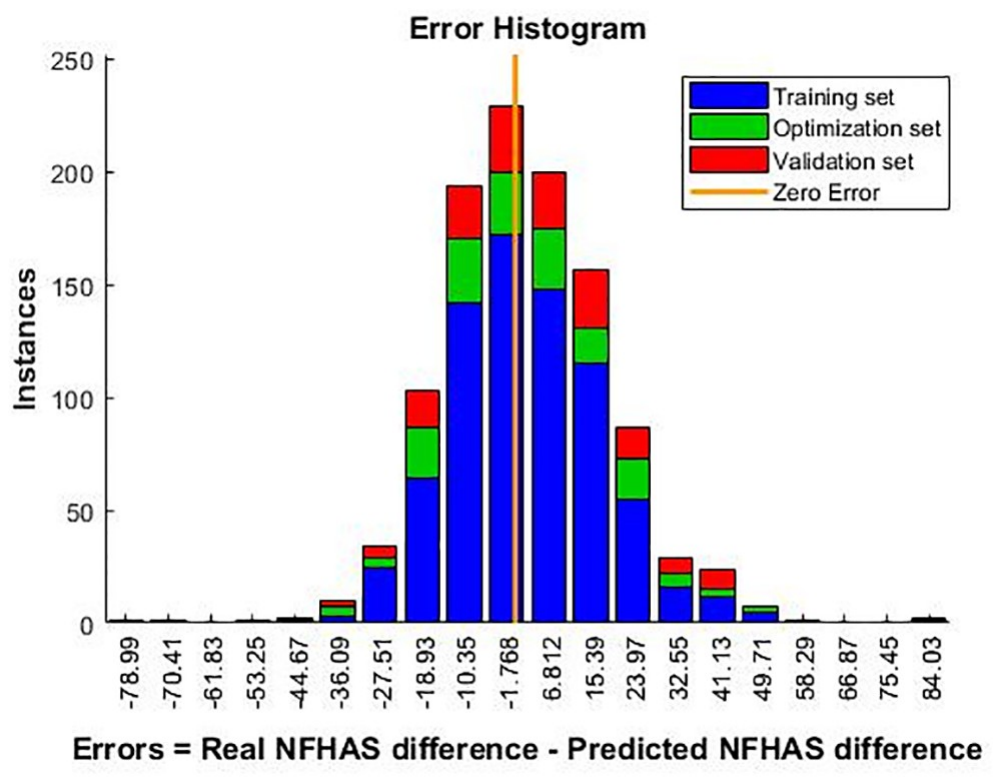
Error histogram from the performance of the ANN.

The distribution of error in the histogram is similar for all the sets used both in training and validation. Regression error is high for all the training sets. The ANN showed a greater tendency to overestimate the NFHAS difference (Fig 4).

### ANN performance on samples not used for training

**Fig 5:**
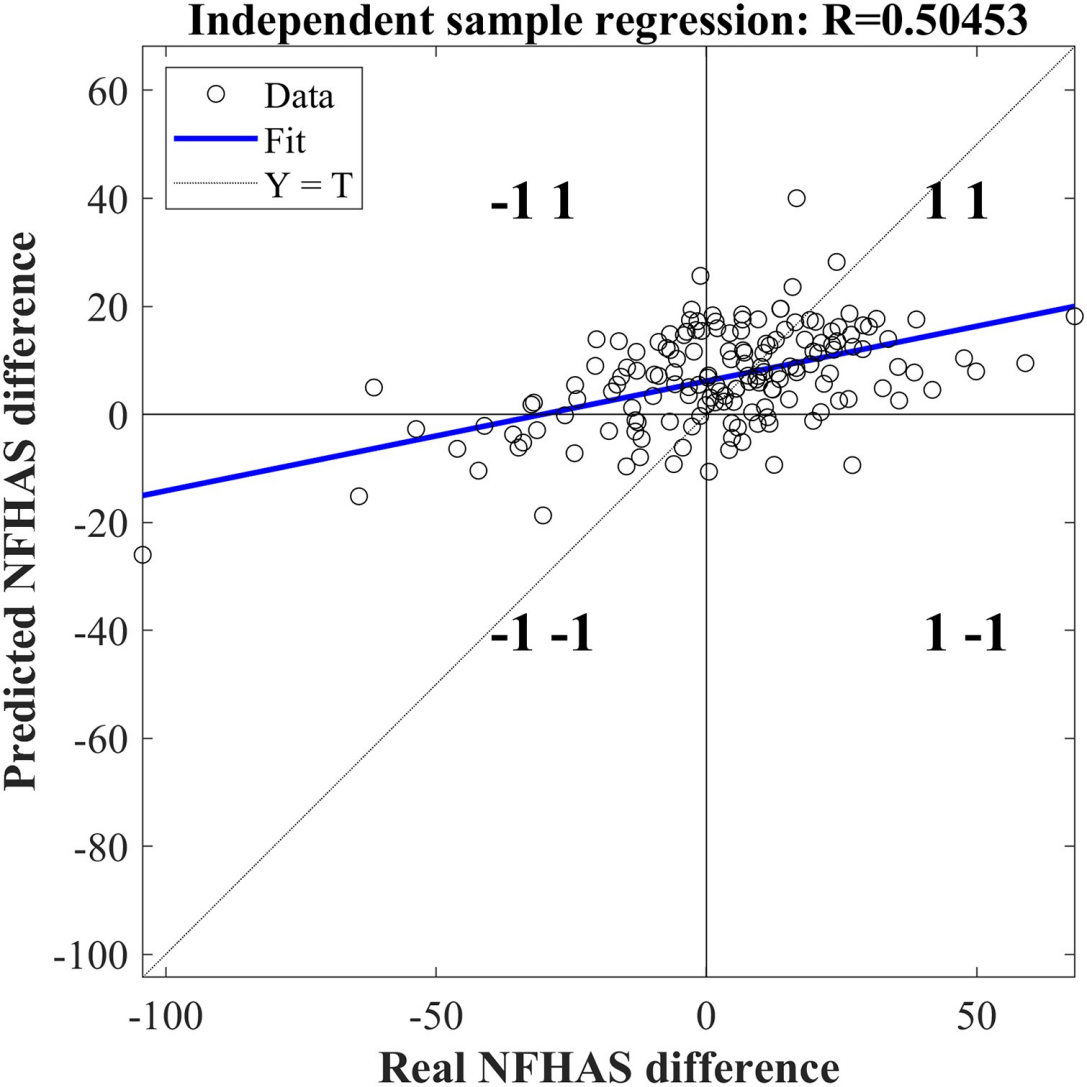
Regression with the predicted difference of the NFHAS and the actual difference.

The generalisation performance of the ANN was tested against a subsample of patients not used in the training. This comparison resulted in a medium correlation with R = 0.5. The final MSE of this ANN was 243.29 (Fig 5). With these results 46.3% true positives (group 1 1), 5.5% true negatives (group -1–1), 20.9% false positives(group -1 1) and 27.1% false negatives (group 1–1).

**Fig 6:**
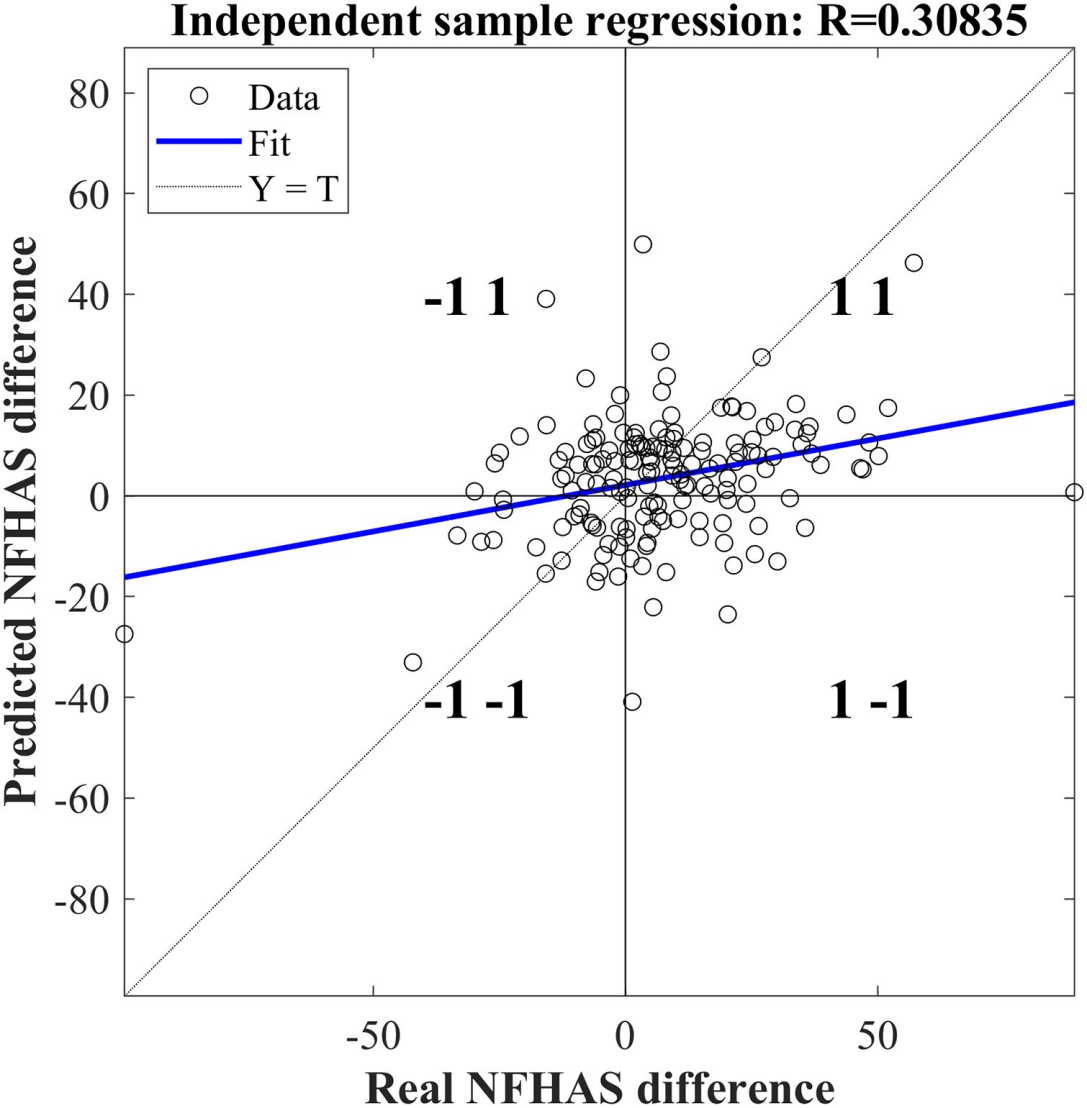
Regression with the predicted difference of the NFHAS and the actual difference on the ANN using PCA.

When PCA is added as pre-processing treatment on the data, the performance of the ANN deteriorates. Correlation drops to R = 0.3 and the MSE increases to 410.36 (Fig 6).

**Fig 7:**
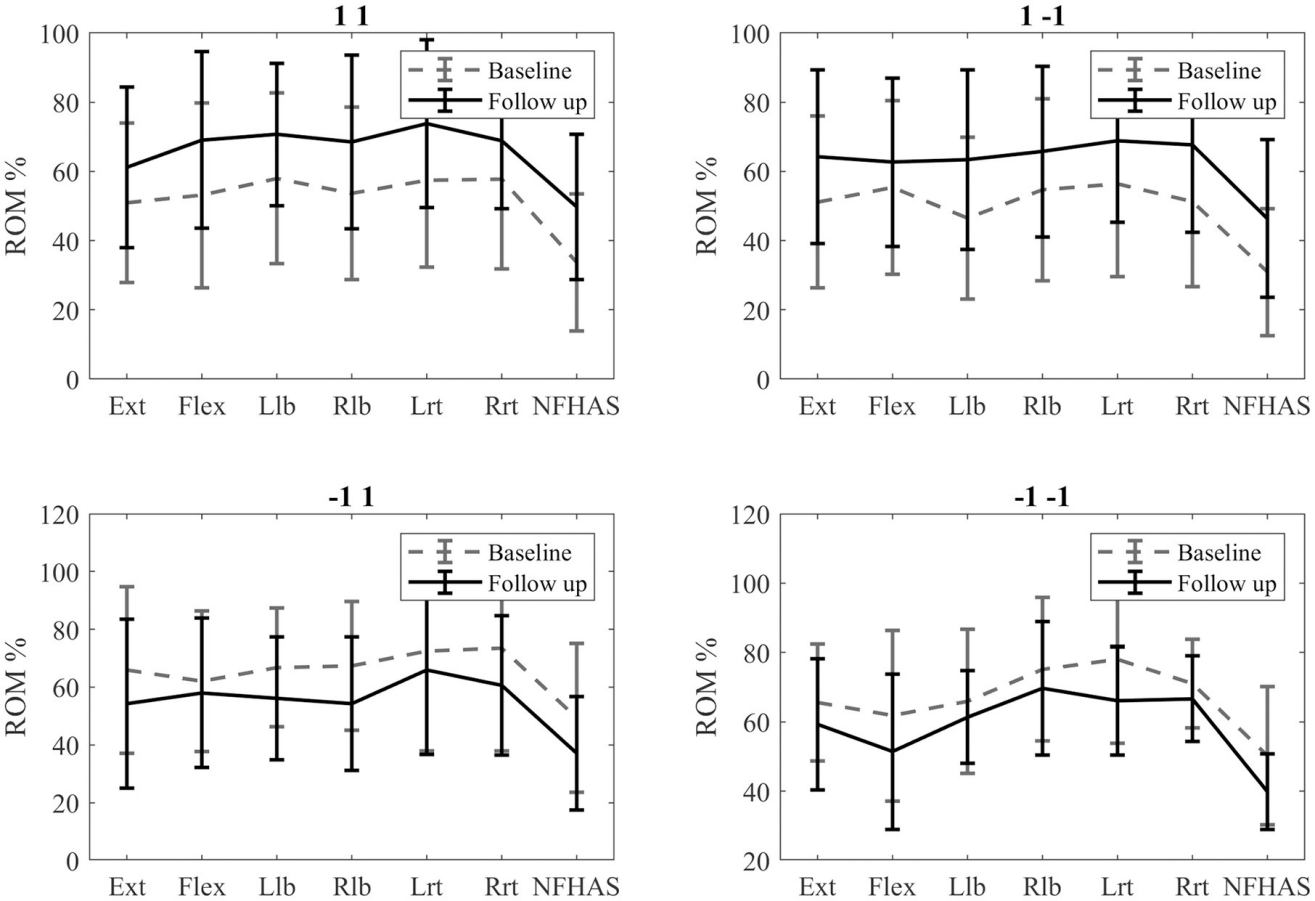
Comparison of the pre- and post-rehabilitation ROM values.

The pre- and post-rehabilitation ROM values in the 1 1 group showed an increase after rehabilitation (Fig 7). However, no significant differences could be found (Table 1).

**Table 1:** Comparison of the pre and post rehabilitation ROM values in the group that improved and was correctly classified as improving (Group 1 1).

	Paired samples t-test results ROM group 1 1						
	Extension Pre vs Post	Flexion Pre vs Post	Left lateral bending Pre vs Post	Right lateral bending Pre vs Post	Left rotation Pre vs Post	Right rotation Pre vs Post	NFHAS Pre vs Post
Mean difference (standard error)	4.18(3.46)	3.22(2.94)	6.39(3.34)	-1.2611(3.46)	3.04(3.99)	3.46(3.38)	2.89(2.35)
p Value	0.2234	0.2694	0.05654	0.7126	0.4403	0.3016	0.2146
CI	-10.9896	-8.9950	-12.9652	-5.5330	-10.8798	-10.1071	-7.5106
	2.6091	2.5480	0.1777	8.0551	4.7813	3.1736	1.7153

The 1–1 group also showed an overall improvement while significant differences could only be found in the left rotation (Table 2, Fig 7).

**Table 2:** Comparison of the pre and post rehabilitation ROM values in the group that improved and was incorrectly classified as not improving (Group 1–1). *Significant at the 0.05 level of statistical significance.

	Paired samples t-test results ROM group 1–1						
	Extension Pre vs Post	Flexion Pre vs Post	Left lateral bending Pre vs Post	Right lateral bending Pre vs Post	Left rotation Pre vs Post	Right rotation Pre vs Post	NFHAS Pre vs Post
Mean difference (standard error)	3.62(5.29)	6.04(5.29)	2.01(4.04)	6.21(4.38)	12.82(5.68)	8.48(4.83)	6.78(3.59)
p Value	0.4826	0.2441	0.6081	0.1514	0.0253*	0.0774	0.0587
CI	-14.0142	-16.4260	-9.9417	-14.8167	-23.9676	-17.9551	-13.8391
	6.7625	4.3284	5.9079	2.3938	-1.6897	0.9858	0.2645

The -1 1 group showed a tendency to decrease the ROM values after rehabilitation (Fig 7). There were significant differences in the right lateral bending, right rotation and NFHAS (Table 3).

**Table 3:** Comparison of the pre and post rehabilitation ROM values in the group that did not improve and was incorrectly classified as improving (Group -1 1). *Significant at the 0.05 level, **Significant at the 0.01 level of statistical significance.

	Paired samples t-test results ROM group -1 1						
	Extension Pre vs Post	Flexion Pre vs Post	Left lateral bending Pre vs Post	Right lateral bending Pre vs Post	Left rotation Pre vs Post	Right rotation Pre vs Post	NFHAS Pre vs Post
Mean difference (standard error)	6.91(4.27)	6.09(3.69)	5.76(3.76)	8.01(4.08)	6.54(3.74)	10.48(5.34)	7.98(4.06)
p Value	0.1034	0.0971	0.1222	0.0301*	0.0793	0.0051**	0.0046**
CI	-15.2867	-13.3318	-13.1462	-15.2159	-13.8948	-17.6428	-13.3638
	1.4652	1.1519	1.6106	-0.8082	0.7991	-3.3289	-2.5970

All the ROM values tended to decrease after rehabilitation in the -1–1 group (Fig 7). Significant differences were only found for the left rotation and NFHAS (Table 4).

**Table 4:** Comparison of the pre and post rehabilitation ROM values in the group that did not improve and was correctly classified as not improving (Group -1–1). *Significant at the 0.05 level, ***Significant at the 0.001 level of statistical significance.

	Paired samples t-test results ROM group -1–1						
	Extension Pre vs Post	Flexion Pre vs Post	Left lateral bending Pre vs Post	Right lateral bending Pre vs Post	Left rotation Pre vs Post	Right rotation Pre vs Post	NFHAS Pre vs Post
Mean difference (standard error)	-2.16	11.98	0.17	3.27	13.90	0.94	6.30
p Value	0.7909	0.1687	0.9669	0.5216	0.0001***	0.8773	0.0420*
CI	-16.0239	-30.2590	-9.8397	-14.5344	-18.4837	-14.5355	-12.3138
	20.3491	6.2802	9.4814	7.9890	-9.3259	12.6554	-0.2910

**Fig 8:**
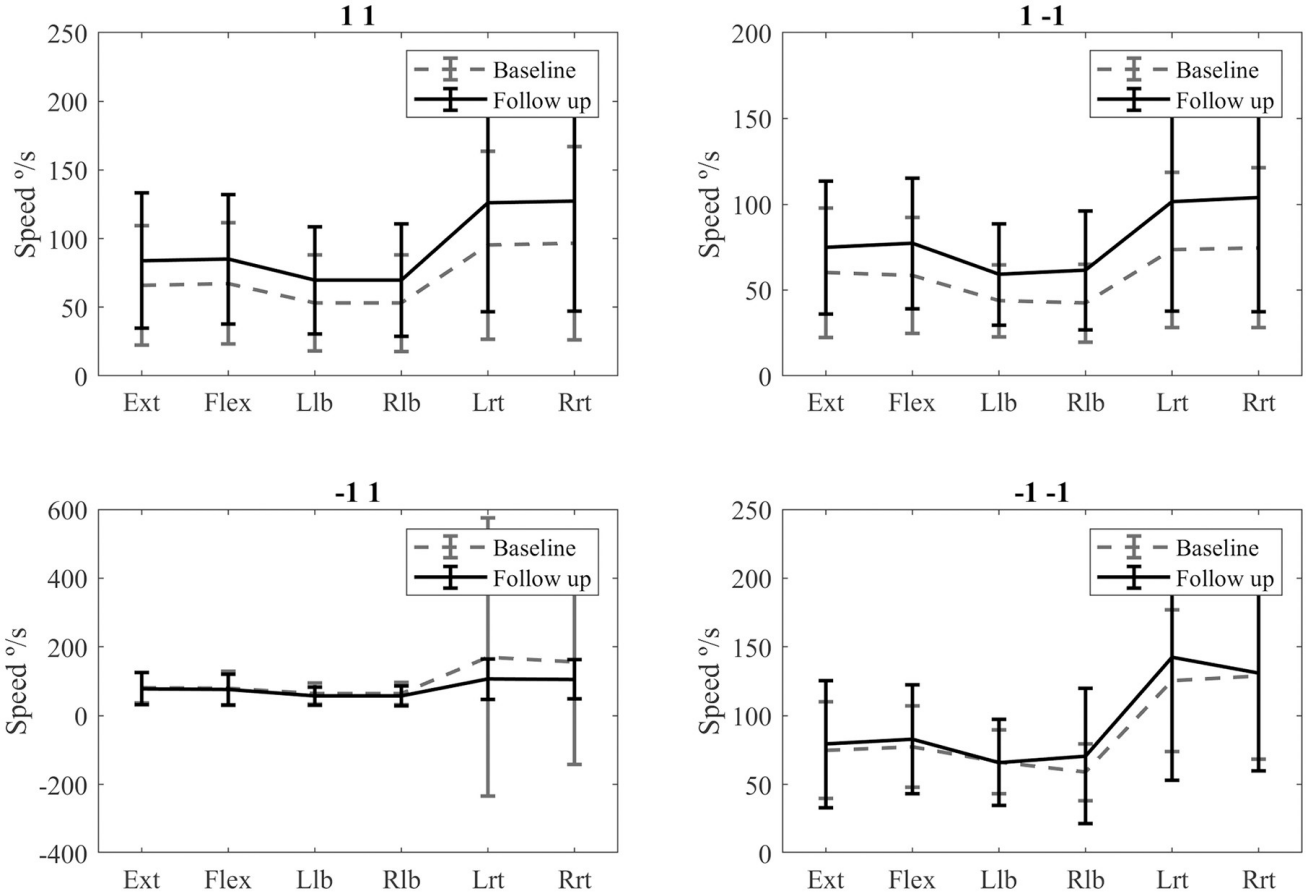
Comparison of the pre and post speed values in the different groups.

All the speed values tend to increase after rehabilitation in the 1 1 group (Fig 8), but no significant differences were found with the paired t-test result (Table 5).

**Table 5:** Comparison of the pre and post rehabilitation speed values in the group that improved and was correctly classified as improving (Group 1 1).

	Paired samples t-test results speed group 1 1					
	Extension Pre vs Post	Flexion Pre vs Post	Left lateral bending Pre vs Post	Right lateral bending Pre vs Post	Left rotation Pre vs Post	Right rotation Pre vs Post
Mean difference (standard error)	7.98(4.33)	7.74(4.44)	2.60(3.08)	4.90(3.27)	10.75(7.43)	10.03(5.96)
p Value	0.0651	0.0810	0.3944	0.1328	0.1458	0.0917
CI	-16.4704	-16.4583	-8.6620	-11.3253	-25.3448	-21.7418
	0.5105	0.9781	3.4535	1.5247	3.8261	1.6679

There were significant differences between the speed scores of the pre- and post-rehabilitation evaluations in the 1–1 group in the left lateral bending, right lateral bending, left rotation, and right rotation (Table 6). All values increase in the post rehabilitation assessment (Fig 8).

**Table 6:** Comparison of the pre and post rehabilitation speed values in the group that improved and was incorrectly classified as not improving (Group 1–1). *Significant at the 0.05 level, **Significant at the 0.01 level, ***Significant at the 0.001 level of statistical significance.

	Paired samples t-test results speed group 1–1					
	Extension Pre vs Post	Flexion Pre vs Post	Left lateral bending Pre vs Post	Right lateral bending Pre vs Post	Left rotation Pre vs Post	Right rotation Pre vs Post
Mean difference (standard error)	13.30(6.57)	12.77(7.44)	9.36(4.07)	10.40(4.48)	28.61(9.63)	33.19(9.73)
p Value	0.0433*	0.0843	0.0225*	0.0218*	0.0041**	0.0012***
CI	-26.1884	-27.3833	-17.3833	-19.2059	-47.4913	-52.2733
	-0.4268	1.8295	-1.4058	-1.6131	-9.7327	-14.1136

There were significant differences between the speed scores of the pre- and post-rehabilitation evaluations in the -1 1 group in the extension, flexion, left lateral bending, right lateral bending, left rotation, right rotation (Table 7). Only the rotation movements show meaningful differences between pre and post values (Fig 8).

**Table 7:** Comparison of the pre and post rehabilitation speed values in the group that did not improve and was incorrectly classified as improving (Group -1 1). **Significant at the 0.01 level, ***Significant at the 0.001 level of statistical significance.

	Paired samples t-test results speed group -1 1					
	Extension Pre vs Post	Flexion Pre vs Post	Left lateral bending Pre vs Post	Right lateral bending Pre vs Post	Left rotation Pre vs Post	Right rotation Pre vs Post
Mean difference (standard error)	19.53(4.92)	16.49(4.71)	10.16(3.94)	13.60(3.77)	34.02(7.08)	32.20(7.74)
p Value	0.0002***	0.0008***	0.0113**	0.0006***	0.0001***	0.0001***
CI	-29.1909	-25.7445	-17.9041	-21.0091	-47.9186	-47.3754
	-9.8807	-7.2502	-2.4224	-6.2057	-20.1232	-17.0297

There were no significant differences between the speed scores of the pre- and post-rehabilitation evaluations (Table 8 and Fig 8).

**Table 8:** Comparison of the pre and post rehabilitation speed values in the group that did not improve and was correctly classified as not improving (Group -1–1).

	Paired samples t-test results speed group -1–1					
	Extension Pre vs Post	Flexion Pre vs Post	Left lateral bending Pre vs Post	Right lateral bending Pre vs Post	Left rotation Pre vs Post	Right rotation Pre vs Post
Mean difference (standard error)	5.83(10.63)	11.72(12.18)	3.70(9.21)	-0.63(9.23)	16.88(18.96)	25.61(19.4)
p Value	0.5367	0.2904	0.6494	0.9376	0.3256	0.1590
CI	-26.6944	-35.6161	-21.7799	-17.4726	-54.0651	-63.6428
	15.0194	12.1609	14.3772	18.7402	20.2979	12.4136

**Fig 9:**
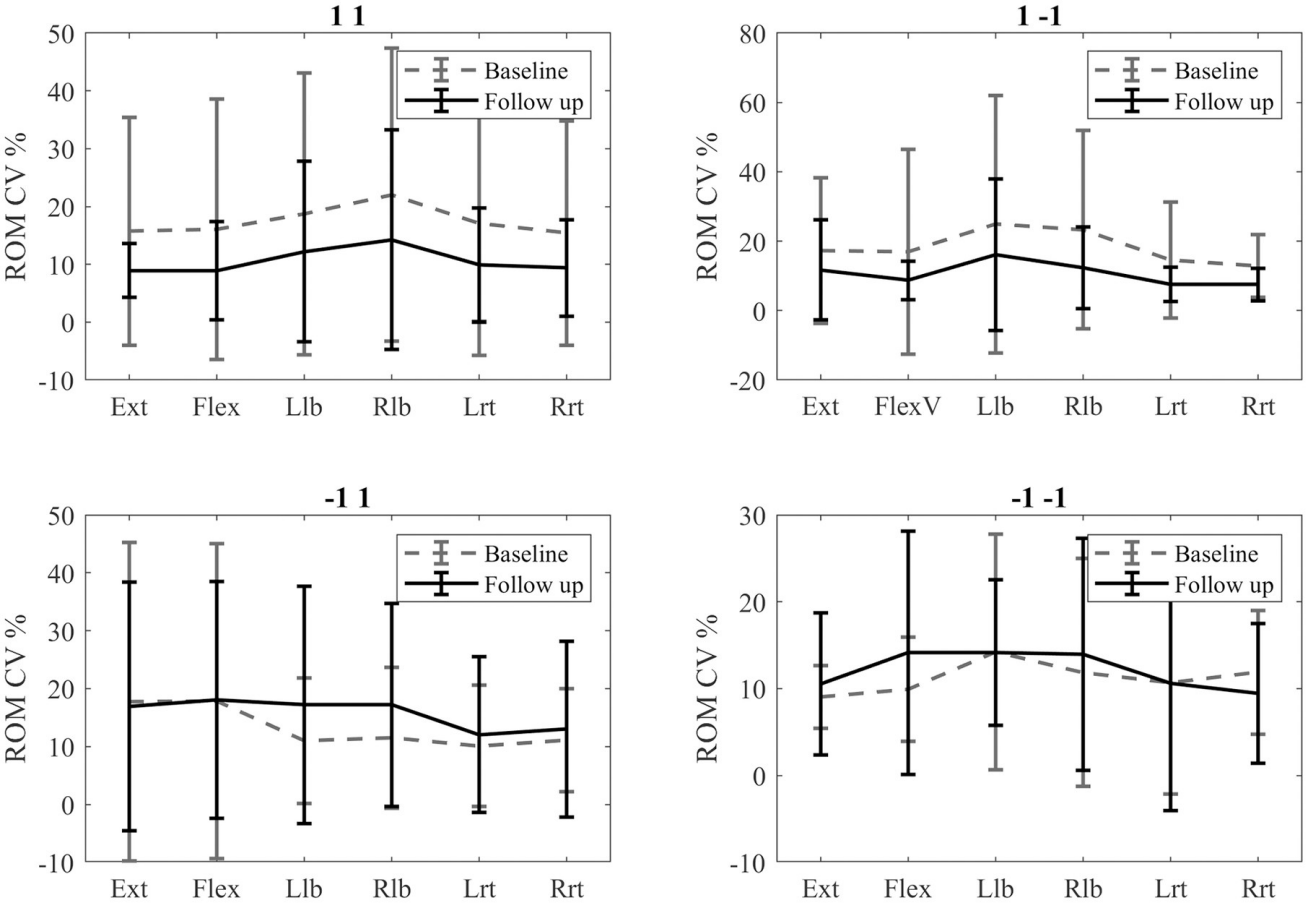
Comparison of the pre- and post-rehabilitation CV ROM values.

The 1 1 group showed a tendency to reduce the ROM CV value after rehabilitation. There were only significant differences in the extension, left rotation, and right rotation (Table 9 and Fig 9).

**Table 9:** Comparison of the pre and post rehabilitation ROM CV values in the group that improved and was correctly classified as improving (Group 1 1). *Significant at the 0.05 level, **Significant at the 0.01 level of statistical significance.

	Paired samples t-test ROM CV group 1 1					
	Extension Pre vs Post	Flexion Pre vs Post	Left lateral bending Pre vs Post	Right lateral bending Pre vs Post	Left rotation Pre vs Post	Right rotation Pre vs Post
Mean difference (standard error)	-8.56(2.87)	1.58(3.03)	-4.50(3.94)	-2.98(3.27)	-5.51(2.56)	-5.31(2.66)
p Value	0.0034**	0.5959	0.2501	0.3583	0.0317*	0.0465*
CI	2.9269	-7.5353	-3.2373	-3.4468	0.4985	0.0842
	14.2026	4.3569	12.2421	9.4126	10.5410	10.5419

The 1–1 group also decreased the ROM CV value after rehabilitation, but significant differences were found only for the flexion movement (Table 10 and Fig 9).

**Table 10:** Comparison of the pre and post rehabilitation ROM CV values in the group that improved and was incorrectly classified as not improving (Group 1–1). *Significant at the 0.05 level of statistical significance.

	Paired samples t-test ROM CV group 1–1					
	Extension Pre vs Post	Flexion Pre vs Post	Left lateral bending Pre vs Post	Right lateral bending Pre vs Post	Left rotation Pre vs Post	Right rotation Pre vs Post
Mean difference (standard error)	-1.17(2.26)	-5.92(2.89)	3.17(3.72)	-1.71(4.52)	-4.76(4.02)	-1.54(5.71)
p Value	0.5943	0.0412*	0.3838	0.6963	0.2279	0.7805
CI	-3.2702	0.2501	-10.4839	-7.1562	-3.1262	-9.6550
	5.6205	11.6080	4.1397	10.5901	12.6573	12.7493

The -1 1 group displayed the opposite behaviour to the last two. Some values of the ROM CV increased after rehabilitation. However, no significant differences were found (Table 11 and Fig 9).

**Table 11:** Comparison of the pre and post rehabilitation ROM CV values in the group that did not improve and was incorrectly classified as improving (Group -1 1).

	Paired samples t-test ROM CV group -1 1					
	Extension Pre vs Post	Flexion Pre vs Post	Left lateral bending Pre vs Post	Right lateral bending Pre vs Post	Left rotation Pre vs Post	Right rotation Pre vs Post
Mean difference (standard error)	-5.23(3.87)	-3.45(2.63)	-0.82(2.4)	-4.55(2.93)	-1.83(1.44)	-4.94(2.84)
p Value	0.1723	0.1848	0.7270	0.1182	0.1973	0.0808
CI	-2.3699	-1.7157	-3.8959	-1.2077	-0.9929	-0.6319
	12.8353	8.6286	5.5404	10.3199	4.6705	10.5307

Significant differences were found between the ROM CV scores of the right rotation between pre- and post-rehabilitation evaluations in the -1–1 group. The figure shows however some of the values of the ROM CV increasing after rehabilitation (Table 12 and Fig 9).

**Table 12:** Comparison of the pre and post rehabilitation ROM CV values in the group that did not improve and was correctly classified as not improving (Group -1–1). *Significant at the 0.05 level of statistical significance.

	Paired samples t-test ROM CV group -1–1					
	Extension Pre vs Post	Flexion Pre vs Post	Left lateral bending Pre vs Post	Right lateral bending Pre vs Post	Left rotation Pre vs Post	Right rotation Pre vs Post
Mean difference (standard error)	-1.38(3.03)	-19.65(12.13)	-3.71(2.59)	-5.00(6.69)	-6.95(3.66)	-3.88(1.67)
p Value	0.6072	0.0933	0.1313	0.4048	0.0565	0.0261*
CI	-4.5793	-4.1441	-1.3801	-8.1161	-0.2445	0.5956
	7.3461	43.4485	8.8041	18.1239	14.1498	7.1757

## Discussion

The performance results obtained from the different tested architectures are in accordance to the established consensus on the optimal number of hidden layers. The ANN with the smallest MSE had just one hidden layer. The optimal number of neurons is also in accordance to existing rules of thumb since it is said that these should range between 1 and the maximum number of input and output neurons. There is no way of knowing a priori which is the optimal number of neurons. The trial and error approach used, helped to determine the optimal number of neurons and hidden layers. The use of PCA as a pre-processing treatment did not result useful. Applying PCA to a set of variables creates new uncorrelated variables, these variables can in turn help to improve the ANN’s performance, however, the results showed that the both the R value form the regression and the MSE worsened when PCA was applied. As such, PCA was not applied as a pre-processing treatment in the final ANN.

Early identification of patients who recover poorly from WAD would help in the specific management of each case. However, the definition of recovery from WAD is still not well established [3]. Many risk factors have been identified as potentially causing the transition from acute to chronic WAD such as gender, age or self-rated pain intensity [28]. Promising results were obtained by Hendriks et al. using multiple regression models. Their models correctly classified 80% of the patients on average. It is important to notice that the sample of patients was small and mainly included biopsychosocial factors [2]. No investigation could be identified linking kinematic variables as predictors of good or bad recovery.

The present investigation extracted 18 variables from the kinematics of the neck. These kinematic variables are mainly related to functional capability and consistency of movement. Their relationship with pain intensity suggested them as good predictors of good or bad kinematic recovery [1]. The results obtained partially support this thesis. The predictive power of the developed ANN is only moderate, showing a great degree of error. The 0.5 result in the regression analysis demonstrates that the baseline kinematic capabilities of the subject have relation with the expected ROM improvement after rehabilitation. There is however a high degree of uncertainty on how much change there would be. Some reasons can explain this uncertainty. The first reason is the lack of inclusion of other psychosocial factors in the ANN. Psychosocial factors have shown the ability to identify patients at risk of developing chronic WAD. It seems reasonable to think that psychosocial factors could also play a part in the effect of the rehabilitation treatment. Another information that would be important to be included in the ANN is the treatment received during the rehabilitation period. While homogeneity in the received treatment was assumed, it cannot be ensured that reality complied with this expectation. The assessment protocol is standardized and controlled but health professionals have freedom on treatment choice. This freedom of choice handicaps clinical investigation but can help to identify best treatment choices depending on the degree of injury shown by the patient. Including psychosocial factors and treatment as inputs could potentially improve the performance of the ANN.

The behaviour of ROM and speed showed an inverse relationship with the ROM CV. Baseline values according to NFHAS type showed that the first two increase with better kinematic performance while the later decreases. While until NFHAS type 4 the ROM CV increases gradually, the type 5 shows a greater jump, not following a linear increment. Insurance environments have shown signs of negatively influencing patient reported outcomes [38]. Some incentives, such as the financial one, can encourage people to intentionally exaggerate some of their symptoms [39]. The ROM CV quantifies consistency of movement repetition. Feigned efforts have shown a higher ROM CV value than maximal efforts. Recommendations are not to use this value as the only indicative of a feigned effort since the variability of this value across subjects is very large [40]. It is difficult to think that after rehabilitation the movement coordination gets harmed by the treatment and literature does not support this line of thought either [8]. The potential intentional exaggeration of some patients could have further influenced the degree of uncertainty obtained; however, it should not be ruled out the possibility of having witnessed the effect of applying the wrong treatment to a patient. Common treatment for these patients involves manual therapy and the application of diverse electrotherapy techniques. In the other hand, the most recent guidelines recommend different modalities of care depending on the severity of the symptoms [41]. Our results cannot conclude that patients incorrectly predicted are neither intentionally feigning their symptoms nor having been administrated the wrong treatment for their condition, but suggest that an external unobserved factor is causing the patients to show worse outcomes after rehabilitation.

Several limitations should be addressed on this paper. This approach lacks information regarding the psychosocial aspects of the individual. But the chosen variables can be more easily standardized. Furthermore, kinematics such as the ROM get impaired in the presence of pain [13]. If the pain levels are reduced, kinematics should improve in the absence of other limiting factors. Psychosocial data is not collected by default in the clinical environment, and so it was not available for our analysis. Mixing the information presented here with more data concerning biopsychosocial factors could potentially improve the performance of the prediction. Mixing both approaches will be tested in next investigations. The second limitation is that malingering patients cannot be identified objectively. While consistency is worse in feigned movements, it is not possible to establish a threshold to consider a movement as non-consistent [42]. While many researchers are working on this topic, there are no good objective and definitive standards published.

As a conclusion the neural network trained to predict the expected difference in NFHAS between baseline and follow up showed modest results. While the overall performance is moderately correlated, the error of this prediction is still large to use the method in clinical practice. Having achieved moderate correlation is promising nonetheless, and justifies further research exploring the effects of additional clinically relevant factors which could further improve prediction performance.
